# Study of cognitive function in patients with severe asymptomatic carotid artery stenosis by a computerized neuropsychological assessment device

**DOI:** 10.3389/fpsyg.2023.1055244

**Published:** 2023-03-08

**Authors:** Zhongzhou Hu, Kun Zhang, Wei Qiang, Xiangmin Fan, Zhong Chen

**Affiliations:** ^1^Department of Vascular Surgery, The Capital Medical University Affiliated Beijing Anzhen Hospital, Beijing Institute of Heart Lung and Blood Vessel Diseases, Beijing, China; ^2^State Key Laboratory of Computer Science and Beijing Key Lab of Human-Computer Interaction, Institute of Software, Chinese Academy of Sciences, Beijing, China; ^3^School of Computer Science and Technology, University of Chinese Academy of Sciences, Beijing, China

**Keywords:** carotid artery stenosis, no symptom, cognitive function, computerized tests, quantitative evaluation

## Abstract

**Background:**

Carotid stenosis can lead to stroke and cognitive impairment. Moreover, the cognitive function was assessed mostly by paper and pencil cognitive tests. This study aimed to evaluate the impact of severe asymptomatic carotid artery stenosis (SACAS) on cognitive function by a computerized neuropsychological assessment device (CNAD). The diagnostic value of screening SACAS of the CNAD was analyzed.

**Methods:**

There were 48 patients with ≥70% asymptomatic carotid stenosis and 52 controls without carotid stenosis. Duplex ultrasound defined the degree of stenosis. The differences of cognitive function were analyzed between patients and controls. The relationship of scores of cognitive tests and age were analyzed in the linear regression equation. The diagnostic value of CNAD was evaluated by the receiver operating characteristic (ROC) curve.

**Results:**

Stenosis and control subjects had no statistically significant differences in baseline characteristics. Stenosis patients had worse scores for Stroop color-word test (*p* = 0.002), one back test (*p* = 0.013), and identification test (*p* = 0.006) corresponding to attention and executive ability. The analysis of linear regression equation indicated that cognitive scores of stenosis patients declined faster with age, especially for digit span test, Stroop color-word test, one back test and identification test. In analysis of ROC curve, the Stroop color-word test (*p* = 0.002), one back test (*p* = 0.013), and identification test (*p* = 0.006), and comprehensive index of the three tests (*p* = 0.001) had the diagnostic value.

**Conclusion:**

The CNAD has evaluation value and screening value for patients with cognitive impairment and SACAS. But it is necessary to update the CNAD and conduct a study with a bigger sample.

## Introduction

Carotid artery stenosis without incidence of ipsilateral stroke, transient ischemic attack (TIA) is considered asymptomatic. Severe asymptomatic carotid artery stenosis (SACAS) with a 70% or greater diameter reduction is a complication of atherosclerotic cardiovascular disease that occurs in approximately 1.7% of adults (de Weerd et al., 2009). Studies have reported an annual risk of stroke of 3.3% for patients with SACAS (Norris et al., 1991). Moreover, severe carotid stenosis is considered to be an independent risk factor for vascular cognitive impairment (Lal et al., 2017; Ihle-Hansen et al., 2021; Lazar et al., 2021). This does not only apply to dementia cases classified as vascular dementia but does also to Alzheimer’s Disease (Arvanitakis et al., 2016; Heller and Hines, 2017). Therefore, SACAS seriously affects the physical and mental health of the public (Gray et al., 2020). Most of the patients with SACAS missed the best treatment time because they were not detected in time, so it is of great significant to find the patients with SACAS as soon as possible (Qureshi et al., 2007; Gates and Kochan, 2015).

There is a certain relationship between carotid stenosis and cognitive impairment. Moreover, the cognitive impairment caused by carotid stenosis has certain characteristics. For example, it is more strongly related to cognitive functions such as memory, attention, psychomotor speed and executive ability (Mathiesen et al., 2004; Romero et al., 2009; Casas-Hernanz et al., 2012; Lin et al., 2014). Therefore, we imagine that through the cognitive function test between normal people and people with SACAS to find out the specific cognitive impairment pattern and the corresponding diagnostic parameters. Then, people who have SACAS may be identify by the pattern and the diagnostic parameters.

At present, the mild cognitive impairment (MCI) are mostly tested by paper and pencil cognitive tests such as Mini-Mental State Examination (MMSE), Montreal Cognitive Assessment (MoCA) and so on (Folstein et al., 1975; Shulman et al., 2006; De Roeck et al., 2019). However, these paper and pencil cognitive tests have some limitations: (1) The data cannot be stored and played back, due to low digitalization and informationization; (Cullen et al., 2007). (2) The evaluator’s subjectivity maybe influence the evaluation result and lead to a semi-quantitative subjective evaluation (Borson et al., 2019; Nasreddine, 2020). In order to solve above problems, we design an computerized neuropsychological assessment device (CNAD). We aim to compare the differences in cognitive function between the patients with SACAS and the people without carotid stenosis through the CNAD, and to explore the best diagnostic parameters of the system.

## Method

### Patients and study design

This study is a single-center prospective study of patients with severe asymptomatic carotid stenosis (stenosis group) vs. patients without stenosis (control group) at Beijing Anzhen Hospital, Capital Medical University. The recruitment of participants was consecutive from September 2020 to August 2021. Patients were enrolled after approval by the Ethics Committee of Beijing Anzhen Hospital, Capital Medical University, and informed consent was obtained.

Patients recruited to the stenosis group need to meet the following including criteria: (1) Unilateral internal carotid artery stenosis ≥70% according to NASCET criteria (Moneta et al., 1993; Staikov et al., 2002); (2) Age ≥ 50 years but ≤80 years; (3) In the past 6 months, had no significant neurological symptoms, such as stoke, TIA, dementia or depression; (4) Intracranial artery stenosis <50%; (5) No visible structural brain lesions on cranial CT or MRI imaging; (6) Be able to complete all cognitive function tests. Subjects recruited to the control group need to meet the same including criteria as stenosis group, but the first including criterion was replaced with bilateral internal carotid artery without stenosis.

Exclusion criteria for both stenosis group and control group were as follows: (1) Severe systemic or neuropsychiatric disease; (2) History of cognitive impairment; (3) Drugs that affect central nervous system function or cholinesterase inhibitor treatments; (4) Any contraindications to MRI or CT scan.

### Imaging examination

The degree of carotid artery stenosis was assessed by duplex ultrasound in the special vascular examination department of Beijing Anzhen Hospital. Doppler waveforms were acquired at a 60-degree angle between the ultrasound beam and the long axis of the artery. The highest peak systolic and end-diastolic velocity measurement from each index carotid pathway was used to quantify the stenosis. Doppler velocity thresholds to determine the degree of stenosis were according to consensus criteria (≥70% stenosis to near occlusion when internal carotid artery [ICA] peak systolic velocity [PSV] is >230 cm/s; Lal et al., 2017; Zierler et al., 2018). The structural brain lesions on cranial CT or MRI imaging were judged by the radiologist.

### Cognitive testing

The CNAD used in this study was developed by the Institute of Software, Chinese Academy of Sciences. The CNAD quantitatively evaluate cognitive function in multi-dimensions through interactive and digital cognitive testing tools, including digit span test, spatial span test, Stroop color-word test, grammatical reasoning test, one back test, identification test and detection test. The test system runs on the Microsoft Surface Go 2 tablet and needs to be connected to the network during the test. The subjects completed the test by clicking on the screen with their fingers. Cognitive function was assessed automatically by the scoring of the system. The lower the scores in digit span test, spatial span test, Stroop color-word test, grammatical reasoning test, one back test and identification test, and the higher scores in detection test, the more likely the subject is to have cognitive impairment.

Digit span test originated from the digital recall experiment conducted by J. Jacobs in 1887, which is a classical measure of working memory in neuropsychological research and clinical evaluations (Wechsler, 1997a,b; Ostrosky-Solís and Lozano, 2006; Woods et al., 2011). Digit span test requires subjects to memorize the numbers that appeared on the screen and enter them on the keyboard. The number is displayed every second, and after the number display is finished, the page will be automatically turned to the keyboard page. The test starts with two numbers, and the number of numbers increases gradually. Each order of magnitude has two opportunities to answer questions, and the test ends when the opportunity is used up. The score of digit span test is equal to “the highest number of digits that subjects can be remembered.”

Spatial span test was originally developed by Corsi (1972), which can measure the capacity of the visuospatial short-term memory (Kessels et al., 2000). Spatial span test requires subjects to remember the flashed squares in the order, and click on them at the end. The square flashes every other second. Squares are arranged in a format of 3*3. The test starts with a random 3 flashed squares, the number of squares will gradually increase, and there will be two chances to answer questions in each order of magnitude. The test ends when the opportunity is used up. The score of spatial span test is equal to “the maximum number of blocks remembered.”

Stroop color-word test originated from Stroop effect found by Stroop (1935), which is a classical measure of executive function for patients with mild cognitive impairment in the ability of inhibition and control (Bélanger et al., 2010). Stroop color-word test requires subjects to judge the font color of Chinese characters on the screen during 60 s. There are four kinds of Chinese characters: blue “blue,” blue “red,” red “red” and red “blue.” When Chinese characters appear, subjects are asked to judge whether the font color of Chinese characters is “red” or “blue” as soon as possible. The new Chinese characters are displayed 0.5 s after each answer. The score of Stroop color-word test is equal to “the number of correct times minus the number of errors.”

Grammatical reasoning test is a classical paradigm for testing and measuring speech comprehension and reasoning ability (Furnham and Chamorro-Premuzic, 2006). Grammatical reasoning test requires subjects to judge whether the combination of graphics on the screen is consistent with the text description during 60 s. The graphic combination consists of a square and a circle with a certain positional relationship between them. A sentence will appear in the middle of the screen to describe the relationship. If the sentence and the graphic combination express the same meaning, the subjects click “correct,” and if not, click “wrong.” Then, another new question will be displayed 1 s after click. The score of grammatical reasoning test is equal to “the number of correct times minus the number of errors.”

One back test is based on the N-back working memory paradigm, which is an effective method for measuring working memory by reflecting the capacity of the temporary storage and manipulation of remembered information (Owen et al., 2005). One back test requires subjects to judge whether it is the same as the previous number based on the number currently displayed during 60 s. Starting from the second number, the subjects can make a judgment by clicking “same” or “different.” The new number will be displayed 1 s after the click operation. The score of one back test is equal to “the number of correct times minus the number of errors.”

Identification test requires the subjects to concentrate and maintain their attention, perform the task as soon as possible and accurately as possible, and involve the functions of attention and executive function (Belleville et al., 2007). Identification test requires subjects to determine whether the color of the current square is red. The square has a total of two colors, one at random at a time, and the subjects complete the judgment and click “Yes” or “No” for 0.5 s before refreshing the question. The score of identification test is equal to “the number of correct times minus the number of errors.”

Detection test requires subjects to be alert to an object and respond to specific stimuli. The response speed and accuracy of the psychomotor are related to psychological processes such as perception and attention (Levinoff et al., 2005), and can be used to identify diseases such as cognitive and mood disorders (Jiang et al., 2019). Detection test requires subjects to determine whether the current animal was a dog, and if so, click it immediately. The display time of each animal picture is 1.25 s. If there is a click action within this time interval, the picture will disappear immediately. The time interval between the two animal pictures is 1 s. The score of detection test is calculated as “the average number of click errors x 0.1 s for all point-to-dog reaction time and all response time skipping ducks (unified 1.25 s).”

**Figure 1:**
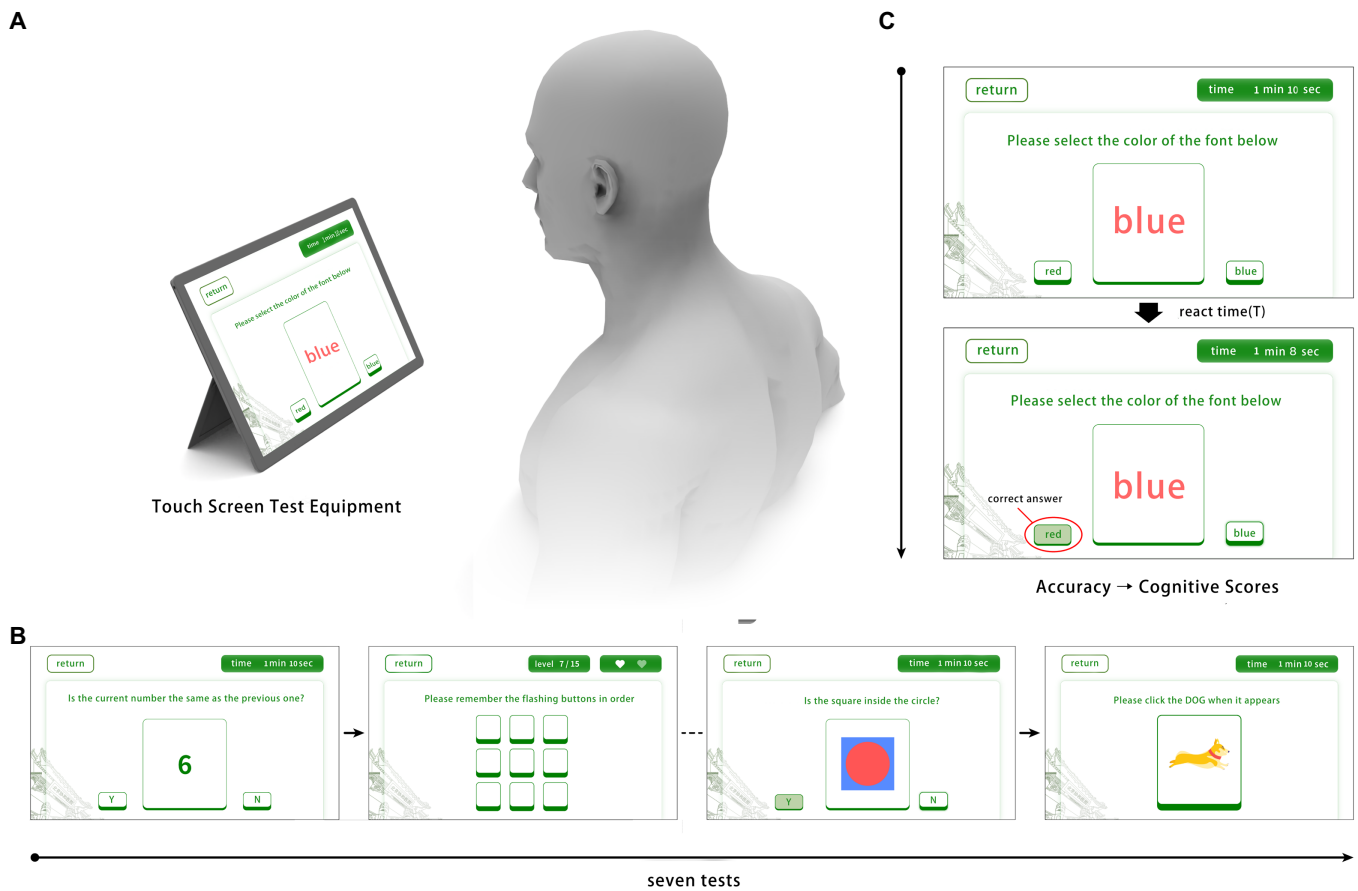
Sample images of actual screens and usage of the CNAD. **(A)** Test schematic diagram; **(B)** Four of seven cognitive tests; **(C)** Scoring criteria and related parameters of testing tasks.

All participants were required to complete all cognitive tests. Testing was conducted in a quiet room and administrated by a master’s-level technician, under the administered in accordance with standardized procedures. The total testing time varied modestly among participants from 20 to 30 min because of interindividual variability in completion time for tests without specific time limits. Sample images of actual screens and usage of the CNAD were showed in Figure 1.

### Statistical analysis

Continuous variables with normal distribution were presented as mean ± standard deviation. Continuous variables with skewed distribution were presented as median (25% quantile, 75% quantile). Categorical variables were presented as frequency (n) or percentage (%). Independent samples *t*-tests or Mann–Whitney *U* tests were used to evaluate baseline clinical characteristics and cognitive scores of stenosis vs. control groups. Contingency table analyses using the χ^2^ statistic were used to determine whether frequencies of some baseline clinical characteristics differed between the groups. To assess the relationship between cognitive function and age, the linear regression analysis was performed between cognitive test scores and age. To explore the best diagnostic parameters of the CNAD, diagnostic tests and receiver operating characteristic (ROC) curves were performed. Data analysis was performed using SPSS version 20 (IBM Corp.Armonk.NY) and MedCalc Statistical Software version 18.2.1 (MedCalc Software bvba, Ostend, Belgium; http://www.medcalc.org; 2018). Significant was set at *p* < 0.05.

## Results

### Clinical characteristics

A total of 105 subjects were recruited in this study. One was excluded for color blindness, one for major hypoacusis, three declined to participate. This resulted in 48 stenosis patients and 52 controls. The baseline clinical characteristics of patients analyzed are detailed in Table 1. The median age was 66 years (25^th^ percentile: 60.5, 75^th^ percentile: 69.5) in the stenosis group and 58 years (25^th^ percentile: 64, 75^th^ percentile: 69.5) in the control group. The proportion of men was similar in both groups. There were no significant differences in Body Mass Index (BMI), education levels, smoking, alcoholism, hypertension, hyperlipidemia, diabetes, and coronary artery disease between stenosis and control groups.

**Table 1:** Baseline clinical characteristics of subjects.

Risk factor	Total (*n* = 100)	Stenosis (*n* = 48)	Control (*n* = 52)	*p*
Male sex	71 (71.0)	33 (68.8)	38 (73.1)	0.634
Age, years	66 (60，69.75)	66 (60.5，69.5)	58 (64，69.5)	0.280
BMI	25.6 ± 3.5	25.0 ± 3.6	26.1 ± 3.3	0.129
Education				0.503
Primary school or below	12 (12.0)	7 (14.6)	5 (9.6)	
Middle school	58 (58.0)	29 (60.4)	29 (55.8)	
College or above	30 (30.0)	12 (25.0)	18 (34.6)	
Smoking	29 (29.0)	11 (22.9)	18 (34.6)	0.198
Alcoholism	14 (14.0)	9 (18.8)	5 (9.6)	0.188
Hypertension	69 (69.0)	35 (72.9)	34 (65.4)	0.416
Hyperlipidemia	89 (89.0)	44 (91.7)	45 (86.5)	0.413
Diabetes	31 (31.0)	17 (35.4)	14 (26.9)	0.359
CAD	68 (68.0)	36 (75.0)	32 (61.5)	0.149

BMI, body mass index; CAD, coronary artery disease.

Categorical variables are presented as number (%). Continuous variables are presented as median (25^th^ percentile, 75^th^ percentile) or mean ± standard deviation (SD).

### Cognitive function results

All patients and control subjects completed all cognitive function testing (Table 2). Stenosis group performed significantly worse on Stroop color-word test (*p* = 0.002), one back test (*p* = 0.013) as well as identification test (*p* = 0.006). There was no significant difference in performance for tests related to digit span (*p* = 0.754), spatial span (*p* = 0.108), grammatical reasoning (*p* = 0.467), detection test (*p* = 0.116).

**Table 2:** The results of cognitive function test between stenosis group and control group.

Name of test	Stenosis	Controls	*p*
Digit span test	6.0 (5.0,7.0)	5.0 (5.0,7.0)	0.754
Spatial span test	5.0 (4.0,5.0)	5.0 (4.0,6.0)	0.108
Stroop color-word test	26.5 (22.5,28.0)	28.0 (26.0,32.0)	0.002
Grammatical reasoning test	5.0 (2.0,7.0)	5.0 (2.0,7.5)	0.467
One back test	19.5 (15.0,24.0)	22.0 (19.0,25.0)	0.013
Identification test	40.0 (37.0,45.0)	44.0 (40.0,48.0)	0.006
Detection test^a^	916.0 (857.0,1000.5)	893.0 (835.5,974.5)	0.116

a For the detection test, lower scores reflect better performance.

In order to explore the relationship between cognitive function and age, the linear regression analysis between cognitive scores and age was conducted in stenosis group and control group separately. The outcomes indicated that in digit span test (stenosis: *p* = 0.043 and controls: *p* = 0.009), Stroop color-word test (stenosis: *p* = 0.021 and control: *p* = 0.017), one back test (stenosis: *p* = 0.023 and controls: *p* = 0.018) and identification test (stenosis: *p* = 0.001 and control: *p* = 0.001) the linear regression equation was established simultaneously in both stenosis group and control group (Table 3). The absolute value of the regression coefficient of the stenosis group was larger than that of the control group in digit span test, Stroop color-word test, one back test， and identification test. Therefore, the cognitive function of the stenosis group declined more significantly with the subjects getting older, especially in identification tests (stenosis: *b* = −0.595 vs. controls: *b* = −0.369).

**Table 3:** The linear regression analysis between cognitive scores and age in stenosis group and control group separately.

Name of test	Stenosis			Controls		
	*p* ^a^	*b* ^b^	*a* ^c^	*p* ^a^	*b* ^b^	*a* ^c^
Digit span test	0.043	−0.082	11.256	0.009	−0.065	10.118
Spatial span test	0.029	−0.038	7.22	0.690	−0.007	5.530
Stroop color-word test	0.021	−0.249	6.877	0.017	−0.223	42.496
Grammatical reasoning test	0.106	−0.068	4.444	0.015	−0.141	13.721
One back test	0.023	−0.344	40.865	0.018	−0.198	34.360
Identification test	0.001	−0.595	78.517	0.001	−0.369	66.426
Detection test	0.057	6.342^d^	674.395	0.221	5.922^d^	469.246

a In the results of variance analysis of linear regression equation, when *p* < 0.05, it can be considered that there is a linear regression relationship between cognitive scores and age.

b b is the regression coefficient of the linear regression equation.

c a is the constant of the linear regression equation.

d For the detection test, lower scores reflect better performance. Therefore, the regression coefficient is a positive number.

**Figure 2:**
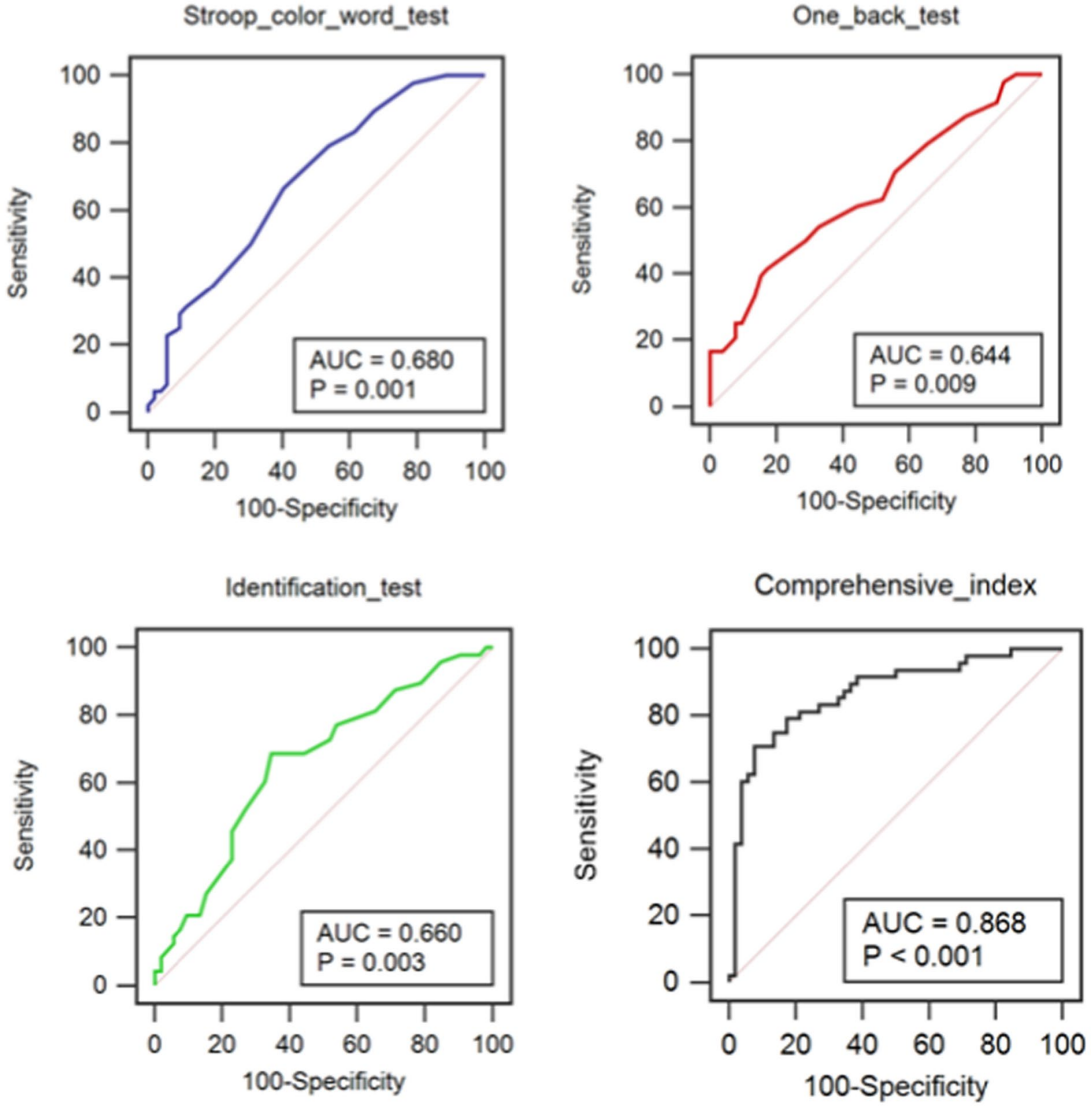
ROC curves of Stroop color-word test, identification test and comprehensive index.

An evaluation of the diagnosis test was performed to explore the value of the CNAD for the diagnosis of SACAS. In the analysis of ROC curve, the area under the ROC curve of 7 cognitive tests shown that Stroop color-word test (*p* = 0.002), one back test (*p* = 0.013) and identification test (*p* = 0.006) had the diagnostic value (Table 4). Therefore, the Stroop color-word test, one back test and identification test were integrated to get the comprehensive index. Moreover, the comprehensive index also had diagnostic value (*p* = 0.001; Table 4). According to the ROC curves (Figure 2) and the comparison of ROC curves using Hardey & McNeil method, these four indices had no significant differences in diagnostic ability. Therefore, the optimal diagnostic cutoff point was calculated for Stroop color-word test, one back test, identification test and comprehensive index. The optimal diagnostic cutoff point is 27 for Stroop color-word test. When the score of Stroop color-word test is no more than 27, subjects are likely to have severe carotid stenosis. The Youden index is 0.2628, the sensitivity is 66.67%, the specificity is 59.62%, and the area under the ROC curve is 0.680, so the diagnostic ability of Stroop color-word test is relatively low. The same is true of one back test (cutoff point ≤18, Youden index = 0.2436, sensitivity = 41.67%, specificity = 82.69%, area = 0.644)，identification tests (cutoff point ≤42, Youden index = 0.3413, sensitivity = 68.75%, specificity = 65.38%, area = 0.660) and comprehensive index (cutoff point >51.563%, Youden index = 0.6314, sensitivity = 70.83%, specificity = 92.31%, area = 0.868). When the score of Stroop color-word test is less than 26, the score of one back test is less than 19 and the score of identification test is less than 46, the comprehensive index reaches to the best optimal cutoff point. The diagnostic specificity and sensitivity of comprehensive index are, respectively, up to 93.2 and 70.83%.

**Table 4:** Area under the ROC curve of 7 cognitive tests and the comprehensive index.

Test Result Variable(s)	Area	*p*	Asymptotic 95% CI^a^	
			Lower bound	Upper bound
Digit span test	0.518	0.759	0.403	0.633
Spatial span test	0.588	0.128	0.477	0.700
Stroop color-word test	0.680	0.002	0.577	0.784
Grammatical reasoning test	0.542	0.469	0.429	0.655
One back test	0.644	0.013	0.536	0.752
Identification test	0.660	0.006	0.553	0.767
Detection test	0.591	0.116	0.480	0.703
Comprehensive index^b^	0.868	0.001	0.615	0.815

a CI, confidence interval.

b Comprehensive index refers to the diagnosis index combined Stroop color-word test and identification test.

## Discussion

In this study, the CNAD showed the characteristics of quantification, semi-automation and traceability. In this way, the subjects can even install the computerized cognitive evaluation system on their tablet PCs to test themselves in their own homes. In addition, without the participation of professionals, it can ensure that the test outcomes are more objective. The CNAD can automatically collect and analysis the related parameters during testing, and the evaluation of the cognitive function can be displayed immediately at the end of the test. These advantages cannot be possessed by the traditional paper and pencil cognitive tests.

Cognitive impairment may be caused by others factors (Sabia et al., 2019). However, in this study, there was no significant difference in sample size and baseline characteristics between the stenosis group and the control group. The results of the cognitive foundation tests showed that there was a statistical difference in the scores of Stroop color-word test，one back test and Identification test between the stenosis group and the control group, but there was no significant difference in other tests. The Stroop color-word test mainly assesses the executive ability. The one back test mainly assesses working memory. The Identification test mainly assesses the attention and executive ability. The other four tests are related to visuospatial short-term memory, speech comprehension and reasoning ability, and response speed and accuracy of the psychomotor. Therefore, according to the outcomes of this study, it can be preliminary inferred that the cognitive functions about attention, working memory and executive ability are significantly decreased in patients with SACAS than normal people without carotid artery stenosis. However, there was no significant difference in cognitive function about visuospatial short-term memory, speech comprehension and reasoning ability, and response speed and accuracy of the psychomotor between two groups. This result is in line with the results of previous studies (Lal et al., 2017; Lazar et al., 2021).

The scores of seven tests and age were analyzed with the linear regression analysis in both stenosis group and control group. The results showed that the scores of digit span test, Stroop color-word test, one back test and identification test decreased with age in both groups, but the score of detection test increased with age. In addition, in these five tests, the absolute value of the regression coefficient of the stenosis group was larger than that of the control group. Therefore, the cognitive function of people with severe asymptomatic carotid stenosis decreased faster than those without carotid stenosis, especially in working memory, attention and executive function (Johnston et al., 2004). However, this conclusion needs to be further assessed by a longitudinal study.

Stroop color-word test, one back test and identification test were identified to have the value of diagnosing severe asymptomatic carotid stenosis according to the outcomes of ROC curve analysis, but their sensitivity and specificity were low. The comprehensive index of Stroop color-word test and identification test could improve the specificity of diagnosis, but the sensitivity was relatively low. Two solutions were proposed. First, new cognitive tests will be added in the computerized cognitive function test system, such as psychomotor speed test, delayed memory test and so on, in order to identify other indicators with more diagnostic value (Era et al., 2011; Abadie and Camos, 2019). Second, multiple diagnostic indicators will be combined to further improve the diagnostic sensitivity and specificity of the comprehensive index (Breton et al., 2019).

### Limitations

The sample size of this study was small. Therefore, more sample size would been needed to obtain more credible and more valuable results.

### Conclusion

The outcomes of this study showed that the cognitive function of patients with severe asymptomatic carotid stenosis is lower than that of normal people, especially in terms of working memory, attention and executive ability. The CNAD has evaluation value and screening value for patients with cognitive impairment and severe asymptomatic carotid stenosis. However, it is necessary to conduct a study with a bigger sample.

## Data availability statement

The original contributions presented in the study are included in the article/supplementary material, further inquiries can be directed to the corresponding author.

## Ethics statement

The studies involving human participants were reviewed and approved by the Ethics Committee of Beijing Anzhen Hospital, Capital Medical University. The patients/participants provided their written informed consent to participate in this study.

## Author contributions

ZH: study design and synthesis analysis. KZ: data collection and analysis. WQ: data process and software development. XF: software development. ZC: study design and manuscript reversion. All authors contributed to the article and approved the submitted version.

## Conflict of interest

The authors declare that the research was conducted in the absence of any commercial or financial relationships that could be construed as a potential conflict of interest.

## Publisher’s note

All claims expressed in this article are solely those of the authors and do not necessarily represent those of their affiliated organizations, or those of the publisher, the editors and the reviewers. Any product that may be evaluated in this article, or claim that may be made by its manufacturer, is not guaranteed or endorsed by the publisher.

## References

[ref1] Abadie M. Camos V. (2019). False memory at short and long term. J. Exp. Psychol. Gen. 148, 1312–1334. doi: 10.1037/xge0000526, PMID: 30550338

[ref2] Arvanitakis Z. Capuano A. W. Leurgans S. E. Bennett D. A. Schneider J. A. (2016). Relation of cerebral vessel disease to Alzheimer's disease dementia and cognitive function in elderly people: a cross-sectional study. Lancet Neurol. 15, 934–943. doi: 10.1016/S1474-4422(16)30029-1, PMID: 27312738 PMC4969105

[ref3] Bélanger S. Belleville S. Gauthier S. (2010). Inhibition impairments in Alzheimer's disease, mild cognitive impairment and healthy aging: effect of congruency proportion in a Stroop task. Neuropsychologia 48, 581–590. doi: 10.1016/j.neuropsychologia.2009.10.021, PMID: 19879885

[ref4] Belleville S. Chertkow H. Gauthier S. (2007). Working memory and control of attention in persons with Alzheimer's disease and mild cognitive impairment. Neuropsychology 21, 458–469. doi: 10.1037/0894-4105.21.4.458 17605579

[ref5] Borson S. Sehgal M. Chodosh J. (2019). Monetizing the MoCA: what now? J. Am. Geriatr. Soc. 67, 2229–2231. doi: 10.1111/jgs.16158, PMID: 31478562

[ref6] Breton A. Casey D. Arnaoutoglou N. A. (2019). Cognitive tests for the detection of mild cognitive impairment (MCI), the prodromal stage of dementia: meta-analysis of diagnostic accuracy studies. Int. J. Geriatr. Psychiatry 34, 233–242. doi: 10.1002/gps.5016, PMID: 30370616

[ref7] Casas-Hernanz L. Garolera M. Badenes-Guia D. Cejudo-Bolivar J. C. Royo J. Aguilar M. (2012). The effect of carotid occlusion in cognition before endarterectomy. Arch. Clin. Neuropsychol. 27, 879–890. doi: 10.1093/arclin/acs075, PMID: 23070315

[ref8] Corsi P. M. (1972).Human memory and the medial temporal region of the brain. J Doctoral Dissertation Mcgill University.

[ref9] Cullen B. O'Neill B. Evans J. J. Coen R. F. Lawlor B. A. (2007). A review of screening tests for cognitive impairment. J. Neurol. Neurosurg. Psychiatry 78, 790–799. doi: 10.1136/jnnp.2006.095414, PMID: 17178826 PMC2117747

[ref10] De Roeck E. E. De Deyn P. P. Dierckx E. Engelborghs S. (2019). Brief cognitive screening instruments for early detection of Alzheimer's disease: a systematic review. Alzheimers Res. Ther. 11:21. doi: 10.1186/s13195-019-0474-3, PMID: 30819244 PMC6396539

[ref11] de Weerd M. Greving J. P. de Jong A. W. Buskens E. Bots M. L. (2009). Prevalence of asymptomatic carotid artery stenosis according to age and sex: systematic review and metaregression analysis. Stroke 40, 1105–1113. doi: 10.1161/strokeaha.108.532218, PMID: 19246704

[ref12] Era P. Sainio P. Koskinen S. Ohlgren J. Härkänen T. Aromaa A. (2011). Psychomotor speed in a random sample of 7, 979 subjects aged 30 years and over. Aging Clin. Exp. Res. 23, 135–144. doi: 10.1007/BF03351077, PMID: 21743291

[ref13] Folstein M. F. Folstein S. E. McHugh P. R. (1975). "Mini-mental state". A practical method for grading the cognitive state of patients for the clinician. J. Psychiatr. Res. 12, 189–198. doi: 10.1016/0022-3956(75)90026-6 1202204

[ref14] Furnham A. Chamorro-Premuzic T. (2006). Personality, intelligence and general knowledge. Learn. Individ. Differ. 16, 79–90. doi: 10.1016/j.lindif.2005.07.002

[ref15] Gates N. J. Kochan N. A. (2015). Computerized and on-line neuropsychological testing for late-life cognition and neurocognitive disorders: are we there yet? Curr. Opin. Psychiatry 28, 165–172. doi: 10.1097/YCO.0000000000000141, PMID: 25602241

[ref16] Gray V. L. Goldberg A. P. Rogers M. W. Anthony L. Terrin M. L. Guralnik J. M.. (2020). Asymptomatic carotid stenosis is associated with mobility and cognitive dysfunction and heightens falls in older adults. J. Vasc. Surg. 71, 1930–1937. doi: 10.1016/j.jvs.2019.09.020, PMID: 31699511 PMC7196504

[ref17] Heller S. Hines G. (2017). Carotid stenosis and impaired cognition: the effect of intervention. Cardiol. Rev. 25, 211–214. doi: 10.1097/CRD.0000000000000139 28786896

[ref18] Ihle-Hansen H. Ihle-Hansen H. Sandset E. C. Hagberg G. (2021). Subclinical carotid artery atherosclerosis and cognitive function: a mini-review. Front. Neurol. 12:705043. doi: 10.3389/fneur.2021.705043, PMID: 34393982 PMC8355501

[ref19] Jiang H. Chen S. Wang L. Liu X. (2019). An investigation of limbs exercise as a treatment in improving the psychomotor speed in older adults with mild cognitive impairment. Brain Sci. 9:277. doi: 10.3390/brainsci9100277, PMID: 31623274 PMC6827026

[ref20] Johnston S. C. O'Meara E. S. Manolio T. A. Lefkowitz D. O'Leary D. H. Goldstein S.. (2004). Cognitive impairment and decline are associated with carotid artery disease in patients without clinically evident cerebrovascular disease. Ann. Intern. Med. 140, 237–247. doi: 10.7326/0003-4819-140-4-200402170-00005, PMID: 14970146

[ref21] Kessels R. P. van Zandvoort M. J. Postma A. Kappelle L. J. de Haan E. H. (2000). The Corsi block-tapping task: standardization and normative data. Appl. Neuropsychol. 7, 252–258. doi: 10.1207/S15324826AN0704_8, PMID: 11296689

[ref22] Lal B. K. Dux M. C. Sikdar S. Goldstein C. Khan A. A. Yokemick J.. (2017). Asymptomatic carotid stenosis is associated with cognitive impairment. J. Vasc. Surg. 66, 1083–1092. doi: 10.1016/j.jvs.2017.04.038 28712815

[ref23] Lazar R. M. Wadley V. G. Myers T. Jones M. R. Heck D. V. Clark W. M.. (2021). Baseline cognitive impairment in patients with asymptomatic carotid stenosis in the CREST-2 trial. Stroke 52, 3855–3863. doi: 10.1161/STROKEAHA.120.032972, PMID: 34433306 PMC8608701

[ref24] Levinoff E. J. Saumier D. Chertkow H. (2005). Focused attention deficits in patients with Alzheimer's disease and mild cognitive impairment. Brain Cogn. 57, 127–130. doi: 10.1016/j.bandc.2004.08.058, PMID: 15708202

[ref25] Lin C. J. Tu P. C. Chern C. M. Hsiao F. J. Chang F. C. Cheng H. L.. (2014). Connectivity features for identifying cognitive impairment in presymptomatic carotid stenosis. PLoS One 9:e85441. doi: 10.1371/journal.pone.0085441, PMID: 24454868 PMC3893296

[ref26] Mathiesen E. B. Waterloo K. Joakimsen O. Bakke S. J. Jacobsen E. A. Bønaa K. H. (2004). Reduced neuropsychological test performance in asymptomatic carotid stenosis: the Tromsø study. Neurology 62, 695–701. doi: 10.1212/01.wnl.0000113759.80877.1f, PMID: 15007116

[ref27] Moneta G. L. Edwards J. M. Chitwood R. W. Taylor L. M. Jr. Lee R. W. Cummings C. A.. (1993). Correlation of North American Symptomatic Carotid Endarterectomy Trial (NASCET) angiographic definition of 70 to 99% internal carotid artery stenosis with duplex scanning. J. Vasc. Surg. 17, 152–159. doi: 10.1067/mva.1993.42888, PMID: 8421332

[ref28] Nasreddine Z. S. (2020). MoCA test mandatory training and certification: what is the purpose? J. Am. Geriatr. Soc. 68, 444–445. doi: 10.1111/jgs.16267, PMID: 31792923

[ref29] Norris J. W. Zhu C. Z. Bornstein N. M. Chambers B. R. (1991). Vascular risks of asymptomatic carotid stenosis. Stroke 22, 1485–1490. doi: 10.1161/01.str.22.12.1485 1962321

[ref30] Ostrosky-Solís F. Lozano A. (2006). Digit span: effect of education and culture. Int. J. Psychol. 41, 333–341. doi: 10.1080/00207590500345724

[ref31] Owen A. M. McMillan K. M. Laird A. R. Bullmore E. (2005). N-back working memory paradigm: a meta-analysis of normative functional neuroimaging studies. Hum. Brain Mapp. 25, 46–59. doi: 10.1002/hbm.20131, PMID: 15846822 PMC6871745

[ref32] Qureshi A. I. Alexandrov A. V. Tegeler C. H. Hobson R. W. 2nd Baker J. D. Hopkins L. N.. (2007). Highlights of the guidelines for screening of extracranial carotid artery disease: a statement for healthcare professionals from the multidisciplinary practice guidelines committee of the american society of neuroimaging; cosponsored by the society of vascular and interventional neurology. J. Endovasc. Ther. 14, 469–474. doi: 10.1177/152660280701400406, PMID: 17696620

[ref33] Romero J. R. Beiser A. Seshadri S. Benjamin E. J. Polak J. F. Vasan R. S.. (2009). Carotid artery atherosclerosis, MRI indices of brain ischemia, aging, and cognitive impairment: the Framingham study. Stroke 40, 1590–1596. doi: 10.1161/STROKEAHA.108.535245, PMID: 19265054 PMC2705324

[ref34] Sabia S. Fayosse A. Dumurgier J. Schnitzler A. Empana J. P. Ebmeier K. P.. (2019). Association of ideal cardiovascular health at age 50 with incidence of dementia: 25 year follow-up of Whitehall II cohort study. BMJ 366:l4414. doi: 10.1136/bmj.l4414, PMID: 31391187 PMC6664261

[ref35] Shulman K. I. Herrmann N. Brodaty H. Chiu H. Lawlor B. Ritchie K.. (2006). IPA survey of brief cognitive screening instruments. Int. Psychogeriatr. 18, 281–294. doi: 10.1017/S1041610205002693, PMID: 16466586

[ref36] Staikov I. N. Nedeltchev K. Arnold M. Remonda L. Schroth G. Sturzenegger M.. (2002). Duplex sonographic criteria for measuring carotid stenoses. J. Clin. Ultrasound 30, 275–281. doi: 10.1002/jcu.10078, PMID: 12116107

[ref37] Stroop J. R. (1935). Studies of interference in serial verbal reactions. J. Exp. Psychol. 18, 643–662. doi: 10.1037/h0054651

[ref38] Wechsler D. WAIS-III: Wechsler adult intelligence scale administration and scoring manual. (1997a). Psychological Corporation.

[ref39] Wechsler D. WMS-III: Wechsler memory scale administration and scoring manual. (1997b). Psychological Corporation.

[ref40] Woods D. L. Kishiyamaa M. M. Lund E. W. Herron T. J. Edwards B. Poliva O.. (2011). Improving digit span assessment of short-term verbal memory. J. Clin. Exp. Neuropsychol. 33, 101–111. doi: 10.1080/13803395.2010.493149, PMID: 20680884 PMC2978794

[ref41] Zierler R. E. Jordan W. D. Lal B. K. Mussa F. Leers S. Fulton J.. (2018). The Society for Vascular Surgery practice guidelines on follow-up after vascular surgery arterial procedures. J. Vasc. Surg. 68, 256–284. doi: 10.1016/j.jvs.2018.04.018, PMID: 29937033

